# 超高效液相色谱-串联质谱法测定阿胶中的驴皮源成分

**DOI:** 10.3724/SP.J.1123.2021.02003

**Published:** 2021-11-08

**Authors:** Liping GONG, Feng SHI, Shufang SU, Qiangsheng XIE, Ruiqing XIAN, Baojian HANG, Yanxia ZHAO

**Affiliations:** 山东省食品药品检验研究院, 国家药品监督管理局胶类产品质量评价重点实验室, 山东 济南 250101; Shandong Institute for Food and Drug Control, National Medical Products Administration(NMPA) Key Laboratory for Quality Evaluation of Gelatin Products, Jinan 250101, China; 山东省食品药品检验研究院, 国家药品监督管理局胶类产品质量评价重点实验室, 山东 济南 250101; Shandong Institute for Food and Drug Control, National Medical Products Administration(NMPA) Key Laboratory for Quality Evaluation of Gelatin Products, Jinan 250101, China; 山东省食品药品检验研究院, 国家药品监督管理局胶类产品质量评价重点实验室, 山东 济南 250101; Shandong Institute for Food and Drug Control, National Medical Products Administration(NMPA) Key Laboratory for Quality Evaluation of Gelatin Products, Jinan 250101, China; 山东省食品药品检验研究院, 国家药品监督管理局胶类产品质量评价重点实验室, 山东 济南 250101; Shandong Institute for Food and Drug Control, National Medical Products Administration(NMPA) Key Laboratory for Quality Evaluation of Gelatin Products, Jinan 250101, China; 山东省食品药品检验研究院, 国家药品监督管理局胶类产品质量评价重点实验室, 山东 济南 250101; Shandong Institute for Food and Drug Control, National Medical Products Administration(NMPA) Key Laboratory for Quality Evaluation of Gelatin Products, Jinan 250101, China; 山东省食品药品检验研究院, 国家药品监督管理局胶类产品质量评价重点实验室, 山东 济南 250101; Shandong Institute for Food and Drug Control, National Medical Products Administration(NMPA) Key Laboratory for Quality Evaluation of Gelatin Products, Jinan 250101, China; 山东省食品药品检验研究院, 国家药品监督管理局胶类产品质量评价重点实验室, 山东 济南 250101; Shandong Institute for Food and Drug Control, National Medical Products Administration(NMPA) Key Laboratory for Quality Evaluation of Gelatin Products, Jinan 250101, China

**Keywords:** 超高效液相色谱-串联质谱, 特征性多肽, 阿胶, 胶原蛋白, ultra-high performance liquid chromatography-tandem mass spectrometry (UHPLC-MS/MS), marker peptide, Asini Corii Colla, collagen

## Abstract

近年来由于驴皮资源短缺,阿胶价格大幅度上涨,市场上出现了大量以马、骡、猪、牛皮等熬制而成的假胶,导致阿胶质量的参差不齐,严重扰乱了市场,急需高效准确的检测方法提升阿胶品质。该研究采用超高效液相色谱-串联质谱技术,建立了阿胶中驴皮源成分的检测方法。样品加水溶解后,于37 ℃下经胰蛋白酶酶解,产生驴源性特征肽段,以0.1%甲酸乙腈溶液和0.1%甲酸水溶液作为流动相进行梯度洗脱,单次分析时间10 min,在电喷雾正离子(ESI^+^)模式下进行多反应监测(MRM),同位素内标法定量。驴源多肽A1、A2在50~1250 μg/L范围内线性关系良好,相关系数(*r*)均大于0.996,方法定量限(*S/N*=10)为20 mg/kg,在300、600、900 mg/kg 3个添加水平上驴源多肽A1、A2的回收率范围为103.2%~108.3%,各加标水平平行测定结果的相对标准偏差(RSD)为1.0%~3.0%,完全能够满足实际样品检测需求。对29批不同生产企业的阿胶进行测定,结果表明,不同企业生产的阿胶中驴源多肽A1、A2的含量之和存在差异,含量为0.096%~0.180%,平均值为0.151%,提示驴源多肽A1、A2含量较低的阿胶生产厂家应注重皮源质量,改进生产工艺,以提升产品质量。该方法操作简便,结果可靠,重现性好,可用于阿胶中驴皮源成分的测定。

## Abstract (en)

In recent years, due to the shortage of donkey skin resources, the price of Asini Corii Colla has seen a rapid increase. Consequently, fake gelatin prepared from horse, mules, pig, and cow skin has appeared in the market, resulting in unreliable quality of Asini Corii Colla. Therefore, there is an urgent need to develop an efficient and accurate method for improving the quality of Asini Corii Colla. Ultra-high performance liquid chromatography-tandem mass spectrometry (UHPLC-MS/MS) was used to determine the donkey skin components in Asini Corii Colla. Accordingly, 0. l g of the evenly mixed sample was weighed and placed in a 50 mL volumetric flask; then, 1% ammonium bicarbonate solution was added to dissolve the sample, and the solution was diluted to the scale. Precisely 1.00 mL of the solution was extracted into a 5 mL volumetric flask, followed by the addition of 1.0 mL trypsin solution and 100 μL mixed internal standard working solution. This mixture was diluted to the scale using 1% ammonium bicarbonate solution, shaken, and placed in an incubator for 16 h to induce enzymolysis at a constant temperature of 37 ℃. The mixture was subsequently removed from the incubator, cooled to ambient temperature, filtered through a 0.22 μm membrane, and analyzed by LC-MS. Separation was performed on an UPLC system with a BEH C18 column (100 mm×2.1 mm, 2.5 μm) under gradient elution using acetonitrile containing 0.1% (v/v) formic acid (B) and water containing 0.1% (v/v) formic acid (A) as the mobile phases at a flow rate of 0.3 mL/min. The column temperature was 30 ℃, and the sample size was 2 μL. The gradient elution conditions are: 0-1 min, 10%B; 1-5 min, 10%B-30%B; 5-5.1 min, 30%B-70%B; 5.1-7 min, 70%B; 7-7.1 min, 70%B-10%B; 7.1-10 min, 10%B. The marker peptides were determined in positive electrospray ionization (ESI ^+^) and multiple reaction monitoring (MRM) modes using the isotopic internal standard method. The optimized enzymolysis conditions were as follows: enzymolysis temperature, 37 ℃; enzymolysis time, 16 h; and amount of enzyme, 1 mL. The two marker peptides showed good linearities in the range of 50 to 1250 mg/L; the correlation coefficients (*r*) were greater than 0.996, and the limits of quantitation (*S/N*=10) were 20 mg/kg. At spiked levels of 300 mg/kg, 600 mg/kg, and 900 mg/kg, the average recovery ratios of the two marker peptides were 103.2% to 108.3%, while the relative standard deviations (RSDs) of 1.0%-3.0%. This method was favorable for testing actual samples. Asini Corii Colla from 29 production companies was detected by this method, and the sum contents of the two marker peptides was different because the production process and raw materials were different. The sum contents of the samples were 0.096% to 0.180% with an average of 0.151%. The developed method is simple, reliable, and reproducible, and it is suitable for detecting the donkey hide components Asini Corii Colla.

阿胶是以马科动物驴的干燥皮或鲜皮经煎煮、浓缩制成的固体胶,其主要成分为驴皮胶原蛋白^[1]^。阿胶的检测方法有差示扫描量热法、凝胶电泳鉴别法、二维相关红外光谱法等分析方法^[2,3,4]^,可分别用于不同来源的阿胶的检测。这些方法都是蛋白质类成分的通用检测方法,专属性不强。

目前动物皮源成分的检测方法主要为荧光定量聚合酶链式反应(PCR)法、液相色谱-串联质谱法等^[5,6,7,8,9,10,11]^。荧光定量PCR法能鉴定马、牛、猪、骆驼、鹿等皮源材料,实现对动物皮源材料的快速检测。阿胶是以驴皮熬制而成,经过熬制过程的阿胶,其所含动物皮源的DNA大部分被破坏,无法获取动物皮源中真实的DNA,因此利用PCR法难以实现阿胶的真伪鉴别。胶原蛋白为不同物种皮的主要成分,经高温熬制后成为不完全水解的肽段,再经胰蛋白酶酶切后可形成物种特异性的多肽。质谱法主要是通过获取动物皮源特征肽段的相对分子质量信息实现蛋白质种类的鉴别^[11,12,13,14,15]^,具有高灵敏度、高准确性、易操作性等优点,可用于阿胶的鉴别。

本研究采用超高效液相色谱-串联质谱(UHPLC-MS/MS)引入同位素内标建立了阿胶中驴皮源成分的检测方法,相比于现有检测方法分析时间短,定量更准确,检测效率提高,可用于阿胶中驴皮源成分的检测。

## 1 实验部分

### 1.1 仪器、试剂与材料

AB SCIEX Triple Quad 5500超高效液相色谱-三重四极杆质谱联用仪(AB公司); KQ-300GDV型恒温数控超声器(昆山市超声仪器有限公司); Vortex-6涡旋振荡器(海门市其林贝尔仪器制造有限公司); FW-80高速万能粉碎机(天津市泰斯特仪器有限公司)。

甲醇、乙腈(色谱纯,美国赛默飞公司),甲酸(色谱纯,美国ACS恩科化学公司),胰蛋白酶(序列分析纯,西格玛公司),去离子水(18.2 MΩ·cm,由Millipore Mili-Q Advantage超纯水系统制得),碳酸氢铵(分析纯,国药集团化学试剂有限公司)。

对照品:驴源多肽A1(C_41_H_68_N_12_O_13_),纯度≥91.5%;驴源多肽A2(C_51_H_82_N_18_O_18_),纯度≥94.2%,购于中国食品药品检定研究院。驴源多肽A1同位素内标(C_41_H_68_N_10_O_13_-^15^N_2_),纯度95.8%;驴源多肽A2同位素内标(C_51_H_82_N_16_O_18_-^15^N_2_),纯度94.6%,由上海强耀生物有限公司合成。

样品:阿胶样品为生产企业提供及市售样品,共29批。

### 1.2 溶液配制

驴源多肽A1、A2标准储备液(1.0 g/L):准确称取驴源多肽A1、A2标准品各10 mg(精确至0.1 mg),分别置于10 mL容量瓶中,用1% (1 g/100 mL,下同)碳酸氢铵溶液溶解并定容至刻度,配制成质量浓度为1.0 g/L的驴源多肽A1、A2标准储备液。

驴源多肽A1、A2内标标准储备液(1.0 g/L):配制过程同驴源多肽A1、A2标准储备液。

混合标准工作溶液(20 mg/L):分别准确吸取驴源多肽A1、A2标准储备液各200 μL于10 mL容量瓶,用1%碳酸氢铵溶液稀释至刻度,配制成质量浓度为20 mg/L的混合标准工作溶液,临用现配。

混合内标工作溶液(10 mg/L):分别准确吸取驴源多肽A1、A2内标标准储备液各100 μL于10 mL容量瓶中,用1%碳酸氢铵溶液稀释至刻度,配制成质量浓度为10 mg/L混合内标工作溶液,临用现配。

标准曲线工作溶液的配制:分别准确移取混合标准工作溶液适量,分别加入一定量混合内标工作溶液,用1%碳酸氢铵溶液稀释,配制成质量浓度为50、100、250、500、1000、1250 μg/L的标准工作溶液(内标质量浓度均为200 μg/L),供UHPLC-MS/MS测定。

胰蛋白酶溶液: 取胰蛋白酶适量,加1%碳酸氢铵溶液溶解,制成每1 mL中含1 mg的溶液。

### 1.3 样品前处理

取样品约10 g,置于粉碎机中粉碎并通过试验筛(孔径0.25~0.45 mm),混匀。称取粉碎好的样品0.1 g(精确至0.0001 g),置于50 mL容量瓶中,加1%碳酸氢铵溶液40 mL,超声处理30 min至样品完全溶解,加1%碳酸氢铵溶液稀释至刻度,摇匀。准确吸取1.00 mL于5 mL容量瓶中,加胰蛋白酶溶液1.0 mL,再加入混合内标工作溶液100 μL,用1%碳酸氢铵溶液稀释至刻度,摇匀,置于恒温箱中,于37 ℃恒温酶解16 h,取出,冷却至室温,经0.22 μm滤膜过滤,待上机测定。

### 1.4 色谱、质谱条件

液相色谱条件:色谱柱为Waters XBridge BEH C18(100 mm×2.1 mm, 2.5 μm)。流动相A为0.1%甲酸水溶液,B为0.1%甲酸乙腈溶液,梯度洗脱程序:0~1 min, 10%B; 1~5 min, 10%B~30%B; 5~5.1 min, 30%B~70%B; 5.1~7 min, 70%B; 7~7.1 min, 70%B~10%B; 7.1~10 min, 10%B。流速为0.3 mL/min,柱温30 ℃,进样量2 μL。

质谱条件:电喷雾离子源(ESI)正离子扫描模式,多反应监测;离子源温度:550 ℃。定性、定量离子对、锥孔电压、碰撞能量见表1。

**表1:** 驴源多肽A1、A2的质谱采集离子信息

Marker peptide	t_R_/ min	Parent ion^#^ (m/z)	Product ion (m/z)	Cone voltage/V	Collision energy/eV
A1	3.23	469.40	712.37^*^	50	16
			783.45	50	15
A1-^15^N2	3.23	470.40	712.40	50	24
A2	3.21	618.50	779.40^*^	52	24
			850.40	52	20
A2-^15^N2	3.21	619.50	779.40	52	30

# Doubly charged ion; * quantitative ion.

## 2 结果与讨论

### 2.1 色谱柱的选择

根据驴源多肽A1、A2的特性,本实验比较了在不同色谱柱BEH Shiled RP C18(100 mm×2.1 mm, 1.7 μm)、XBridge BEH C18(100 mm×2.1 mm, 2.5 μm)、BEH C18(100 mm×2.1 mm, 1.7 μm)上驴源多肽A1、A2的色谱保留行为。结果表明不同色谱柱上驴源多肽A1、A2的保留时间不同,但均能通过调节流动相中有机相的比例,实现驴源多肽A1、A2与基质中干扰组分的有效分离。考虑方法的通用性,推荐使用压力小的XBridge BEH C18(100 mm×2.1 mm, 2.5 μm)为分析色谱柱。

### 2.2 流动相的选择及洗脱条件

选用乙腈-0.1%甲酸水溶液(流动相1)、0.1%甲酸乙腈-0.1%甲酸水溶液(流动相2)两种流动相体系进行试验。结果表明,采用流动相2时得到的驴源多肽A1、A2的离子信号要优于采用流动相1,这是由于有机相中酸的加入,保证了流动相在梯度变化过程中氢离子浓度的稳定性,提高了被分析化合物的离子化效率。因此,本法最终选择0.1%甲酸乙腈溶液-0.1%甲酸水溶液作为流动相。

通过优化流动相的初始比例,使驴源多肽A1、A2的保留时间适中且峰形对称。驴源多肽A1、A2及其同位素内标溶液(50 mg/L)的MRM色谱图见图1。

**图1:**
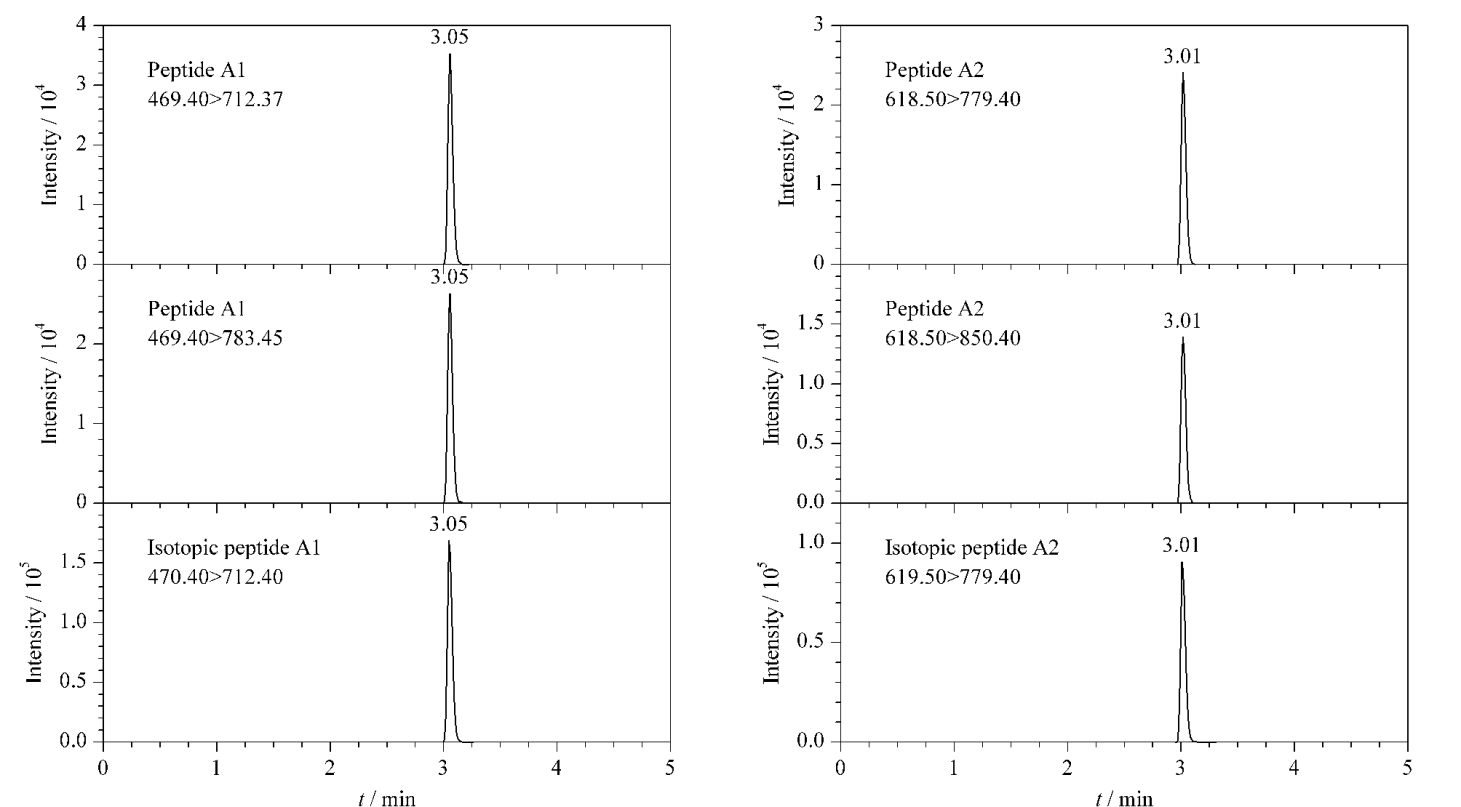
特征肽及其同位素内标溶液的MRM色谱图

### 2.3 质谱参数的优化

将驴源多肽A1、A2标准溶液(1 mg/L)通过蠕动泵(流速10 μL/min)进样,通过一级全扫描找到驴源多肽A1、A2的母离子,再分别以一级母离子通过二级全扫描找到二级碎片离子,并通过不断改变碰撞能使碎片离子响应增强,通过优化获得最佳离子源参数,确定碎裂电压及碰撞能量。

### 2.4 酶解条件的优化

采用胰蛋白酶水解阿胶,适宜的酶解温度为35~40 ℃,根据该酶的特性及使用说明书,本方法采用酶解温度为37 ℃。

酶解时间的考察:固定酶解温度(37 ℃)和酶用量(加胰蛋白酶溶液1.0 mL),考察酶解时间对测定结果的影响,比较了不同酶解时间(0、1、2、4、6、8、16、24、40 h)产生的驴源多肽A1、A2的峰面积,结果见图2。由图2可以看出,随着酶解时间的增长,驴源多肽A1、A2的含量逐渐升高,当酶解时间超过16 h以后,结果趋于稳定,说明酶解反应趋于完全,因此本方法选择酶解时间为16 h。

**图2:**
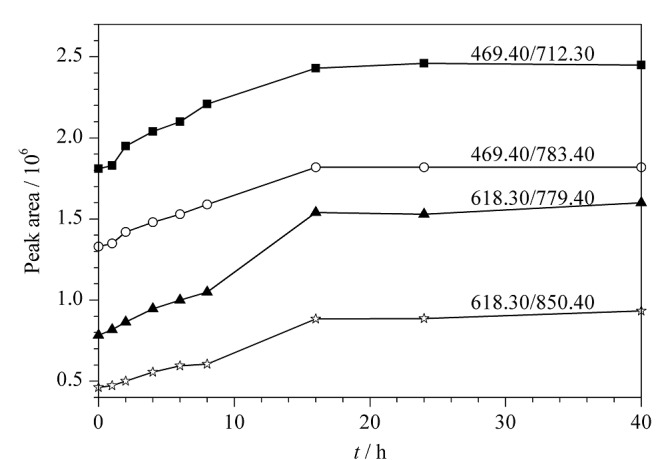
不同酶解时间下监测离子的峰面积

酶的用量:固定酶解温度(37 ℃)和酶解时间(16 h),考察酶的用量对测定结果的影响,结果见图3。由图3可以看出,在胰蛋白酶质量浓度为10 g/L时,酶的用量超过1.0 mL以后,结果趋于稳定,说明当酶的用量为1.0 mL时,足以完全酶解样品,综合考虑到成本等问题,本方法选择酶的用量为1.0 mL。

**图3:**
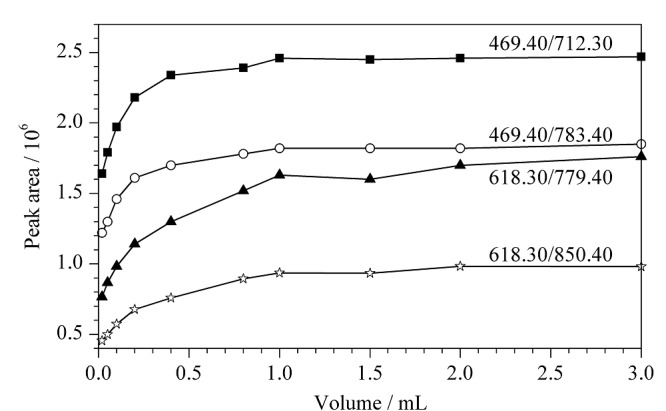
不同酶用量下监测离子的峰面积

### 2.5 定量方式的确定与基质效应评价

采用液相色谱-质谱联用法进行基质复杂样品分析,其复杂基质可能对测定结果产生影响,因此在方法建立时需对基质效应进行评价^[16]^。本方法测定的目标物为阿胶经酶解产生的驴源性多肽,由于无法获得不含驴源性多肽A1、A2的空白基质,因此以牛皮胶代替评估基质效应^[17,18]^。取混合标准工作溶液,按1.2节标准曲线工作溶液的配制过程制备系列纯溶剂标准工作溶液(不含内标);取牛皮胶按照1.3节制得酶解前的样品溶液,以该溶液配制5倍浓度的系列标准工作溶液(不含内标),再按照1.3节样品酶解步骤酶解,作为基质标准工作溶液,分别绘制标准曲线,按文献^[19]^中基质效应计算方法对绝对基质效应进行评估,结果显示驴源多肽A1、驴源多肽A2的绝对基质效应分别是37.5%、39.2%,说明基质效应强,需要消除或降低基质效应。本方法采用同位素内标法消除基质效应,按上述操作重新绘制标准曲线(含内标),再次评估基质效应分别为4.9%、5.2%,基质效应明显降低,表明纯溶剂标准曲线内标法可确保定量结果的准确性。

### 2.6 标准曲线、线性范围及定量限

取系列标准溶液进样,按上述色谱条件测得峰面积,以目标物峰面积与相应内标峰面积的比值为纵坐标*Y*,对照品质量浓度为横坐标*X*,绘制标准曲线,结果表明在50~1250 μg/L范围内,线性关系良好(见表2)。测定的29批样品中驴源多肽A1的含量为216~414 μg/L,驴源多肽A2的含量为293~357 μg/L,所测试样浓度均在标准曲线线性范围内。将标准溶液逐级稀释,以10倍信噪比的峰高对应的质量浓度为定量限,均为10 μg/L,折合到阿胶中含量为20 mg/kg(见表2)。

**表2:** 特征肽的线性范围、回归方程、相关系数(*r*)及定量限

Marker peptide	Regression equation	r	LOQ/ (mg/kg)
A1	Y=4.274×10^3^X+1.259×10^2^	0.9995	20
A2	Y=4.960×10^3^X+1.013	0.9990	20

Linear range: 50-1250 μg/L. *Y*: peak area ratio of quantification and isotope internal standard, *X*: mass concentration, μg/L.

### 2.7 稳定性研究

为研究驴源多肽A1、A2溶液的稳定性,本方法考察了驴源多肽A1、A2标准储备溶液、标准工作溶液及阿胶经酶解产生的驴源多肽A1、A2的稳定性。实验考察了标准储备溶液在-20 ℃下放置1、3、7、10、15、33天的稳定性,结果表明放置10天时浓度开始下降,因此应注意标准储备液的使用时间;分别考察了室温下放置0、1、3、5、7、9、11、14、16、18、20、22、24 h的标准工作溶液(1000 μg/L)及阿胶经酶解后样品溶液中目标肽段的峰面积变化情况,结果表明在被考察的24 h内,驴源多肽A1、A2在标准溶液中峰面积的RSD值分别为3.2%、5.9%,在样品溶液中峰面积的RSD值分别为6.8%、9.1%,稳定性良好。

### 2.8 回收率

本方法通过向样品中添加低、中、高3个浓度水平的驴源多肽A1、A2标准溶液,每个水平进行6次平行试验,计算方法回收率,结果见表3。驴源多肽A1、A2的回收率范围为103.2%~108.3%,各浓度水平平行测定结果的相对标准偏差(RSD)为1.0%~3.0%,完全能够满足实际样品的检测需求。

**表3:** 3个水平下的加标回收率及RSD (*n*=6)

Compound	Spiked/(mg/kg)	Recovery/%	RSD/%
A1	300	105.3	1.0
	600	108.3	3.0
	900	103.2	2.1
A2	300	106.9	1.9
	600	107.0	1.9
	900	106.4	2.0

### 2.9 实际样品测定

采用本方法对不同生产企业以及市售阿胶样品进行测定,得到不同厂家不同工艺共29批次阿胶中驴源多肽A1、A2的含量之和。不同厂家因生产工艺、原料等不同,所测得的食品阿胶中驴源多肽A1、A2的含量之和存在差异,含量为0.096%~0.180%,平均值为0.151%。其中1批试样中驴源多肽A1、A2含量之和小于0.10%;从6批含量较低的阿胶试样中,应用本实验室建立的分析方法^[14]^检出猪皮源、马皮源成分。

## 3 结论

本文建立了UHPLC-MS/MS测定阿胶中驴皮源成分含量的方法,考察了酶的用量、酶解时间,进行了基质效应评估,采用同位素内标法定量,方法准确度高。样品测定结果表明不同生产企业的产品驴源多肽含量差异较大,提示部分生产企业需改进工艺提升产品品质。本方法操作简便,结果可靠,重现性好,可用于阿胶中驴皮源成分的测定。
